# Evaluating Patient Adherence and Persistence to Tyrosine-Kinase Inhibitors for Metastatic Renal Cell Carcinoma: A Retrospective Analysis of Real-World Data

**DOI:** 10.1177/11795549251341877

**Published:** 2025-06-18

**Authors:** Zhaohui Liao Arter, David J Benjamin, Yen Cao, Jorge Farias, Ranjit Kumar Thirumaran, Michael Forsyth, Nataliya Mar, Arash Rezazadeh Kalebasty

**Affiliations:** 1 Division of Oncology and Hematology, University of California Irvine Medical Center, Orange, CA, USA; 2 Hoag Family Cancer Institute, Newport Beach, CA, USA; 3 Medical Affairs, Pfizer Inc., New York, NY, USA

**Keywords:** Tyrosine-kinase inhibitors, renal cell carcinoma, real-world data, adherence, persistence

## Abstract

**Background:**

Combination therapy using an immune checkpoint inhibitor (ICI) and a tyrosine kinase inhibitor (TKI) is the standard first-line treatment for metastatic renal cell carcinoma (RCC). Oral TKIs enhance patient autonomy but require strict adherence and persistence.

**Methods:**

We analyzed adults with metastatic RCC at Chao Comprehensive Cancer Center, receiving at least 2 TKI prescriptions between April 29, 2019, and August 29, 2022, assessing adherence via Medication Possession Ratio (MPR) and Proportion of Days Covered (PDC).

**Results:**

A total of 66 individuals and 849 prescriptions were identified. The mean duration of TKI treatment was 237 days, with a median of 201 days. The mean persistence was 303 days, whereas the median was 233 days. Over 180 days, the median MPR was 83%, whereas the median PDC was 72%. The median variable PDC was 86%, while the median variable MPR was 105%. Asian patients experienced the longest average TKI therapy duration at 319 days, while Hispanic patients had the shortest at 223 days.

**Conclusions:**

We observed a significantly longer median duration of oral TKI therapy (201 days) than the reported national average (< 100 days). This analysis of real-world data reveals that lengthier treatment durations for TKI + ICI combinations are feasible.

## Introduction

In the United States, there are approximately 82 000 new cases of renal cell carcinoma (RCC) and almost 15 000 deaths from RCC each year.^1^ Between 75% and 85% of RCCs exhibit a clear cell histology, with less common subtypes including papillary, chromophobe, and medullary. Systemic therapy options for those patients with relapsed or stage 5 disease are largely comprised of oral targeted therapies such as tyrosine kinase inhibitors (TKIs) and immune checkpoint inhibitor (ICI).^2^ The landscape of cancer treatment has been transformed with the development and advancements of oral therapies. The shift from parenteral to oral therapies enhances the control patients can have over their treatment.^3^ A primary challenge of oral antineoplastic treatment is ensuring patients take their medication as indicated (ie, adherence) and for the recommended duration (ie, persistence)—in this case until disease progression or unacceptable toxicity occurs.^4-6^

Tyrosine kinase inhibitors were a standard of care in the first-line treatment of advanced RCC since the approval of sunitinib in 2006. More recently, the concept of combined therapy with a TKI and ICI has been studied and now added to the therapy options for first-line treatment.^7^ These combination therapies have overlapping adverse events that can occur and add an additional level of complexity to the management of this patient population.^8,9^ Individualizing cancer care through toxicity management and judicious use of dose and schedule modifications has been suggested to improve patient adherence, as well as decrease the risk of treatment failure.^10^

Adherence and persistence are 2 unique constructs that quantify a patient’s medication-taking behavior. Adherence specifically addresses the way a patient takes medication that is consistent with provider recommendations specific to timing, dose, and frequency. Adherence is measured over time and is reported as a percentage. Persistence addresses the duration of time that spans from initiation to discontinuation of therapy in which individuals refill their medications frequently and regularly. There is no overarching term that combines these 2 distinct constructs, and reporting of both provides a more thorough understanding of medication-taking behavior. This understanding may prove essential in optimizing the therapeutic goal(s) of treatment for a medication. The terms non-persistency and discontinuation often overlap in the literature; however, the 2 are not identical. A patient may have a momentary lapse in therapy and resumed at a later time, making the patient nonpersistent, although they have not discontinued therapy.^11-13^

This study aimed to analyze pharmacy dispensing and refill data in order to investigate the adherence and persistence rates of TKIs in combination with ICI therapy for patients with advanced RCC at University of California, Irvine Medical Center (UCI), Chao Comprehensive Cancer Center. The goal was to identify areas for quality improvement and implement solutions to enhance persistence and adherence rates, ultimately leading to improved quality of care.

## Methods

### Data source and study population

Pharmacy dispensing and refill data from April 29, 2019, to August 29, 2022, were acquired from the University of California Health Data Warehouse for the purpose of this analysis. The inclusion criteria for the study consisted of adult patients aged 18 years or older at UCI Chao Comprehensive Cancer Center who were diagnosed with advanced RCC and received at least 2 prescriptions for a Food and Drug Administration (FDA)-approved combination of a TKI (axitinib, cabozantinib, or lenvatinib) as part of a combination regimen with an ICI (avelumab, nivolumab, or pembrolizumab). Patients who were diagnosed with advanced RCC at UCI but received treatment outside of UCI were excluded from the analysis. The de-identified data was then shared with Pfizer Outcomes & Analytics team, who supervised the analysis and obtained the results from the Pfizer Data Analysis Team (PfizerWorks).

### Outcomes

This study focused on 2 primary outcome measures: TKI adherence over a fixed 6-month time period and TKI persistence using the Kaplan-Meier method. Adherence was evaluated using 2 methods: medication possession ratio (MPR) and proportion of days covered (PDC). Secondary outcome measures included estimating TKI adherence over both a variable time period and a fixed 1-year time period using MPR and PDC methods, as well as calculating the average duration of therapy (DOT). In addition, various other indicators related to dispensing and refill patterns were analyzed, such as the monthly total number of prescriptions filled (for more detailed methods, see “Supplementary Methods”).

### Data analysis and statistics

The de-identified patient information, collected for evaluation, was recorded in Microsoft Excel, and subsequently analyzed using a statistical program such as Python. Validated metrics were employed to estimate patient adherence and persistence based on the provided pharmacy dispensing data. Descriptive statistics, including counts and percentages, means and standard deviations, medians, and other appropriate measures, were reported for each variable. To calculate persistency, the Kaplan-Meier method was utilized.

## Results

A total of 66 patients were identified who received at least 2 prescriptions of an FDA-approved TKI in combination with an ICI for advanced RCC, of which 48 (73%) were on axitinib, 19 (29%) were on cabozantinib, and 20 (30%) were on lenvatinib (Table 1). There was a total of 849 TKI prescriptions analyzed with the majority being for axitinib (484, 57%), the rest being for cabozantinib (236, 28%) and lenvatinib (129, 15%), with an average of 13 fills per patient (Table 1). The cohort included 27 (41%) white patients, 26 (39%) Hispanic patients, 10 (15%) Asian patients, 1 (2%) American Indian or Alaska Native patient, and 2 (3%) patients who were classified as other.

**Table 1.** Therapeutic class summary.

	TKI	Axitinib	Cabozantinib	Lenvatinib
# Prescriptions	849	484	236	129
# Patients	66	48	19	20
Average fills	13	10	12	6

Abbreviation: TKI, tyrosine-kinase inhibitors,

### Adherence results

Among the patients who had a minimum follow-up period of at least 6 months, the median MPR was 83.3% (Table 2), and the median PDC was 72.0% (Table 3). The median variable MPR was 105%, while the median variable PDC was 86%. Using the threshold of ⩾ 80% to classify patients as adherent to therapy, 54% of patients as measured by MPR, and 23% of patients using the PDC method were classified as adherent to their TKI at 6 months. For those with a minimum 1-year follow-up, median MPR and PDC rates dropped to 74.0% and 64.0%, respectively. The percentage of patients who achieved an 80% or greater adherence rate within the 1-year minimum follow-up group was 49% as measured by MPR, and 15% as measured by PDC. When stratifying adherence measures by race and ethnicity, Hispanic patients were found to have the lowest mean MPR and PDC at both 6 months (79%, 63%) (Tables 4 and 6) and 1 year (63%, 50%) (Tables 5 and 7). Asians were found to have the highest MPR and PDC rates at both 6 months (100%, 87%) and 1 year (82%, 72%) (Tables 4 to 7).

**Table 2.** Fixed MPR adherence measures.

MPR	6 months	1 year
# Patients	63	51
Mean MPR (%)	82.8	70.7
Median MPR (%)	83.3	74
% Patients < 80%	46	51
% Patients > 80%	54	49
% Patients > 90%	44.4	45.1
% Patients > 95%	41.3	39.2
% Patients > 100%	36.5	25.5

Abbreviation: MRP, medication possession ratio.

**Table 3.** Fixed PDC adherence measures.

PDC	6 months	1 year
# Patients	63	51
Mean PDC (%)	68	58
Median PDC (%)	72	64
% Patients < 80%	40	36
% Patients > 80%	23	15
% Patients > 90%	16	9
% Patients > 95%	11	8
% Patients > 100%	3	0

Abbreviation: PDC, proportion of days covered.

**Table 4.** Six-month MPR by race/ethnicity.

	White	Hispanic	Asian
# Patients	27	26	10
Mean MPR (%)	83%	79%	100%
% Patients < 80%	46.2%	54.2%	20%
% Patients > 80%	53.8%	45.8%	80%

Abbreviation: MRP, medication possession ratio.

**Table 5.** One-year MPR by race/ethnicity.

	White	Hispanic	Asian
# Patients	27	26	10
Mean MPR (%)	77%	63%	82%
% Patients < 80%	40%	64.7%	42.9%
% Patients > 80%	60%	35.3%	57.1%

Abbreviation: MRP, medication possession ratio.

**Table 6.** Six-month PDC by race/ethnicity.

	White	Hispanic	Asian
# Patients	27	26	10
Mean PDC (%)	69%	63%	87%
# Patients < 80%	65.4%	75%	20%
# Patients > 80%	34.6%	25%	80%

Abbreviation: PDC, proportion of days covered.

**Table 7.** One-year PDC by race/ethnicity.

	White	Hispanic	Asian
# Patients	27	26	10
Mean PDC (%)	62%	50%	72%
# Patients < 80%	72%	70.6%	57%
# Patients > 80%	28%	29.4%	42.9%

Abbreviation: PDC, proportion of days covered.

### Duration of therapy results

The mean duration of TKI therapy for all patients was 237 days with a median duration of 201 days (Table 8). Asian patients had the longest duration of TKI therapy with a mean of 319 days, followed by white patients with a mean of 241 days, and Hispanic patients with a mean of 223 days. Median DOT followed a similar pattern with Asian patients having the highest median DOT of 299 days, and Hispanic patients having the lowest median DOT of 150 days (Table 8).

**Table 8.** Duration of therapy (DOT).

	All patients	White	Hispanic	Asian	Other	American Indian or Alaska Native
# Patients (%)	66 (100%)	27 (41%)	26 (39%)	10 (15%)	2 (3%)	1 (2%)
Mean DOT (days)	237	241	223	319	64	-
Median DOT (days)	201	250	150	299	64	-

### Persistency results

**Figure 1.**
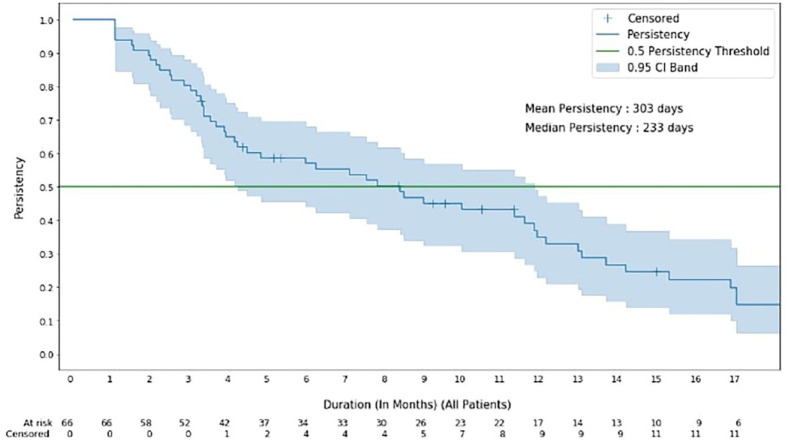
Kaplan-Meier (KM) persistency over the data set.

The mean persistence of TKI treatment for the total cohort of 66 patients was 303 days, whereas the median persistence was 233 days (Figure 1).

## Discussion

In our analysis, we discovered a significantly longer median DOT for TKI + ICI (201 days) compared to the national average (< 100 days) in a small population of mRCC patients from 1 major academic institution.^14,15^ In a retrospective analysis of data from 2007 to 2015 using SEER-Medicare data, the median duration of first-line oral targeted therapy for mRCC was determined to be 71 days.^14^ According to a second retrospective analysis of Veterans Health Administration data from 2010 to 2016, the median DOT for first-line targeted therapy in mRCC was 86 days.^15^ While some other studies have reported longer times to discontinuation,^16-19^ all of the preceding research was conducted prior to the TKI + ICI era. Our study was the first to reflect TKI + ICI adherence patterns based on real-world data.

The adherence to first-line oral targeted therapies observed in this study has important clinical implications. While the treatment landscape for mRCC is rapidly evolving with the addition of immunotherapies,^20,21^ the findings of this study are especially significant because immunotherapies are frequently recommended in combination with targeted therapies, including in the first-line setting (eg, pembrolizumab and axitinib).^2^ Adherence is critical to achieving an optimal clinical response and is unlikely to be the same in practice as it is in trials.

It has been reported that factors such as age, race, comorbidities, and cost are associated with oral anticancer drug adherence.^14,22^ Forgetfulness and lack of a consistent routine are common challenges for patients on oral therapies. Poor understanding of the treatment plan, including the importance of consistent dosing and managing side effects, may further reduce adherence. Patient health status and comorbidities can also play a role, as patients with significant fatigue or gastrointestinal issues may be more likely to skip doses or discontinue therapy. Psychosocial factors such as depression, anxiety, and lack of motivation to continue therapy due to perceived lack of benefit could also contribute to lower adherence rates. In our center, we believe that close follow-up (at least every 2 weeks for the first 3 months) and working with nurse navigator and pharmacist has contributed to patient adherence and persistence (Supplement Figure S1). Patients’ medication adherence can be significantly improved through close follow-up.^23^ Various benefits can happen from close follow-up. (1) Personalized Communication: Regular follow-up appointments allow oncologists to establish a personal connection with patients. They can discuss the importance of medication adherence in managing their condition and address any concerns or misconceptions the patient may have.^24^ (2) Education: During follow-up appointments, an oncologist can provide detailed information about the medications, including their purpose, potential side effects, and how they interact with the patient’s condition. Clear communication can help patients make informed decisions about their treatment.^25^ (3) Monitoring Progress: Close follow-up enables oncologists to monitor the patient’s progress and assess the effectiveness of the prescribed medications. Any adjustments or modifications to the treatment plan can be made based on the patient’s response and feedback.^26^ (4) Addressing Barriers: Patients may encounter various barriers to medication adherence, such as forgetfulness, cost concerns, or difficulty managing multiple medications. Close follow-up allows oncologists to identify these barriers and work collaboratively with the patient to find solutions.^27^ This might involve simplifying the medication regimen, providing reminder tools, or exploring options to reduce costs. (5) Regular Reminders: Scheduled follow-up appointments serve as regular reminders for patients to take their medications. These appointments can help establish a routine and reinforce the importance of adherence. (6) Behavioral Support: During follow-up visits, oncologists can offer behavioral support and strategies to help patients build healthy habits around medication adherence. This might include techniques for incorporating medication-taking into daily routines.^27^ (7) Tracking and Documentation: Regular follow-up allows oncologists to keep track of the patient’s medication history and progress over time. This documentation can help identify patterns and trends in adherence and guide decision-making.^2^ (8) Building Trust: Establishing a strong patient-provider relationship through close follow-up can foster trust and open communication. Patients are more likely to adhere to medications when they feel valued and understood by their health care team. (9). Early Intervention: Close follow-up enables oncologists to detect early signs of non-adherence and intervene promptly. Addressing issues early on can prevent potential complications and setbacks in the patient’s treatment journey. In addition to close follow-up and personalized support, incorporating electronic reminders through patient portals or text messages may further enhance adherence by helping patients establish a consistent routine and reducing forgetfulness. Social media platforms could also serve as tools for patient education and engagement, reinforcing the importance of consistent medication use. Future efforts to improve adherence could explore the integration of electronic health record (EHR)-based alerts, text reminders, and social media engagement as potential strategies to support patients in managing their therapy.^28^

Interestingly, with a limited patient sample size, we also discovered that, in comparison to Hispanic patients, Asians had the best adherence and persistence pattern, followed by whites. Numerous studies have documented racial disparities in RCC prognosis.^29-32^ However, medication adherence was never investigated as a potential outcome-influencing factor. Several factors may contribute to our observation, such as (1) cultural differences surrounding medication adherence with stigma and face-saving.^33^ This might motivate individuals to adhere to prescribed medications to avoid being perceived as having failed to manage their health effectively. (2) Cultural Beliefs about Health. Traditional Asian health philosophies often emphasize balance and harmony in the body.^34^ If individuals believe that medications help restore this balance, they might be more inclined to adhere to treatment. (3) Strong Family Involvement.^35,36^ In many Asian cultures, family plays a central role in decision-making, including health care decisions. Family members might encourage and support medication adherence, contributing to better compliance. To determine whether there is a correlation between race, adherence, education, insurance, and socioeconomic status, additional data will be required.

Overall, our data may provide insight and objective analyses for organizations to internally evaluate areas for improvement or opportunity for patients with advanced RCC. Health care clinicians may be equipped to perform additional evaluations to explore potential causes of unexpected dispensing and refill patterns and implement solutions to improve persistence and adherence rates and ultimately improve the overall quality of care.

## Limitations

It is important to consider several factors when examining persistence that may not have been collected in this analysis. These factors include patient, disease, and treatment characteristics such as age, comorbidities, diagnosis, disease stage, line of therapy, therapy switching, and individual start/stop dates. These factors can significantly impact the interpretation of the results, and therefore, the analysis should be viewed as exploratory and hypothesis generating. The analyses conducted aim to explore trends and statistics based on the available data set, considering its longitudinal nature and the size of the patient population captured. However, it should be noted that patients may have initiated treatment in a different health care system, leading to misclassification of the therapy start date. In addition, patients may have transferred out of the system or obtained prescriptions from other pharmacies, potentially underestimating persistence and adherence.

The calculation of persistence based on the gap between prescription refills may not account for all aspects of patients’ refill behavior, as they are classified as nonpersistent once they exceed the permissible gap. Sensitivity analyses have been conducted to address this limitation. The proposed metrics in this analysis measure medication acquisition rather than actual consumption, and thus, they do not provide certainty regarding whether patients are taking the medication as prescribed in terms of dose and frequency. This is particularly relevant for patients whose medication may be adjusted or paused by their physician to manage their medical condition.^37^

Furthermore, we lack data specifically on the sites of disease. However, we recognize that RCC is a heterogeneous disease, with many patients having hepatic metastases who may not necessarily present with symptoms despite having more concerning disease that warrants aggressive treatment. In addition, the reasons for treatment discontinuation were not collected, and we had no data related to the socioeconomic factors and educational background of the evaluated patient population. We also do not have specific data on adherence for each of the TKIs (axitinib, lenvatinib, and cabozantinib). However, given that all TKIs generally have similar side effect profiles, we believe that there is unlikely to be a significant correlation between adherence and any specific TKI.

It is important to note that the analyses conducted are retrospective and should not be interpreted as representative of future adherence, persistence, or DOT. The results should be interpreted cautiously considering potential missing data, small sample size, and/or insufficient follow-up. Generalization beyond the analyzed patient population and time period should be avoided, and the results should be interpreted within the context of the data and its limitations in measurement.

## Conclusion

Our study is the first, to our knowledge, to assess TKI + ICI adherence patterns based on real-world data. We found a considerably longer median DOT for TKI + ICI combinations at an academic medical center (201 days) compared to the national average (< 100 days before the TKI + ICI era). This suggests that extended treatment durations are achievable in clinical practice. In addition, we discovered that Asians had the best adherence and persistence pattern. Further investigation is needed to explore potential factors, such as clinical monitoring or effective management of adverse effects, that could contribute to increased adherence and persistence to optimize patient outcomes.

## Supplemental Material

sj-docx-1-onc-10.1177_11795549251341877 – Supplemental material for Evaluating Patient Adherence and Persistence to Tyrosine-Kinase Inhibitors for Metastatic Renal Cell Carcinoma: A Retrospective Analysis of Real-World Data Supplemental material, sj-docx-1-onc-10.1177_11795549251341877 for Evaluating Patient Adherence and Persistence to Tyrosine-Kinase Inhibitors for Metastatic Renal Cell Carcinoma: A Retrospective Analysis of Real-World Data by Zhaohui Liao Arter, David J Benjamin, Yen Cao, Jorge Farias, Ranjit Kumar Thirumaran, Michael Forsyth, Nataliya Mar and Arash Rezazadeh Kalebasty in Clinical Medicine Insights: Oncology
