# Supplementary material for: Evaluating Patient Adherence and Persistence to Tyrosine-Kinase Inhibitors for Metastatic Renal Cell Carcinoma: A Retrospective Analysis of Real-World Data
Source: Clin Med Insights Oncol. 2025 Jun 18;19:11795549251341877. doi: 10.1177/11795549251341877 (PMC12177250; doi:10.1177/11795549251341877)

**Supplementary Appendix**

**Methods:**

*Description of Metrics and Method of Calculation*

*Duration of therapy* (DOT) was measured in days and determined by calculating the number of days until a gap of 180 days or more was observed in medication refills for each individual patient. The average DOT was calculated by taking the mean of all the therapy durations for each patient, representing the time until a 180-day gap occurred.
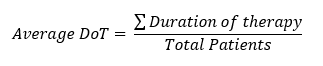


*Medication Possession Ratio* (MPR) – MPR represents the ratio of a patient's total days' supply of therapy to the length of the follow-up period. Both mean and median values were calculated for MPR. Based on the type of follow-up period, MPR was calculated as:

*Fixed MPR* – This ratio was determined by dividing the patient's total days' supply of therapy by the length of the fixed follow-up period. The fixed time period needed to have a post-index follow-up period that was equal to or longer than the period being measured. For instance, a fixed time period of 6 months required a 180-day follow-up period, while a fixed time period of 1 year required a 365-day follow-up period.


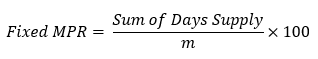


*m:* Number of fixed days in the follow-up period.

Primary outcome measure m = 180 days; Secondary outcome measure m = 365 days

*Variable MPR* – Variable MPR represents the ratio of a patient's total days' supply of therapy to the patient's unique duration of therapy in days. Since each patient's duration of therapy is distinct, it results in a variable follow-up period. Due to the different denominators in the ratios, they could not be directly combined across patients. Instead, the ratios were divided and averaged to obtain an overall adherence value.


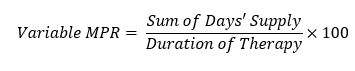


For variable follow up, a gap of 180 days was used to define the end of the follow-up period, which required a runout period of 180 days after the last fill to be observed to mark the end of the variable follow-up period. It is important to note that both variable and fixed MPR values could have exceeded 100%.

*Proportion of Days Covered* (PDC): PDC represents the ratio of days "covered" by the patient on the therapy to the length of the follow-up period. Both mean and median values were calculated for PDC. Based on the type of follow-up period, PDC was calculated as:

*Fixed PDC* – The fixed PDC represented the ratio of days "covered" by the patient on the therapy to the length of the fixed follow-up period in days. The fixed time period required a post-index follow-up period that was equal to or longer than the specific measurement period being calculated. For example, a fixed time period of 6 months necessitated a 180-day follow-up period, while a fixed time period of 1 year required a 365-day follow-up period.


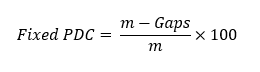


Gaps: The cumulative sum of days in the period *m* where a prescription is not present

*m:* Number of fixed days in the follow-up period.

Primary outcome measure m = 180 days; Secondary outcome measure m = 365 days

*Variable PDC* – The variable PDC is the ratio of days “covered” by the patient on the therapy to the patient’s unique duration of therapy in days.


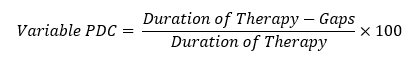


Gaps: The cumulative sum of days in the patient duration of therapy where a prescription is not present

For variable follow up, a gap of 180 days was used to define the end of follow-up period, which required a runout period of 180 days after the last fill to be observed to mark the end of the variable follow-up period. It is worth noting that both variable and fixed PDC values cannot exceed 100%.

*Persistence via Kaplan Meier* – The Kaplan-Meier method was employed to estimate the population persistence curve, also known as the survival curve. This analysis determined the cumulative probability of patients remaining on treatment beyond a specific time point.

To calculate persistence using the Kaplan Meier method, the "event" considered was treatment discontinuation, which was defined as a gap in therapy exceeding the grace period (e.g., 1.5 times the days' supply). The date of discontinuation was determined by the depletion of days' supply from the last prescription filled prior to the therapy gap. Patients who did not discontinue treatment during the study period were right censored at the minimum date at which “discontinuation” could be defined: *Minimum censor date = Last Data Date – Grace period + 1.*

The Kaplan-Meier Survival function was then calculated as a cumulative persistency of continuing patients with each of the preceding time intervals as explained below:

Kaplan-Meier Survival function: *S(t) = (n1-d1)/n1 * (n2-d2)/n2 * ….. * (nk-dk)/nk*

*t = current time (days in this case)*

*nk = number of patients at risk of dropping off the therapy just prior to the time t=k*

*dk = number of patients who drop from therapy at time t=k*

This analysis utilized the Lifelines Library in Python to generate the persistence via Kaplan Meier plots, with the index prescription serving as the starting point for the analysis of persistence.

*Sensitivity Analyses*

Apart from the default value of 1.5 times the days' supply for persistence, sensitivity analyses were performed using two alternative measures for estimating the permissible gap: 1 times the days' supply and 2 times the days' supply.

**Figure S1:** TKI initiation and follow up demonstration


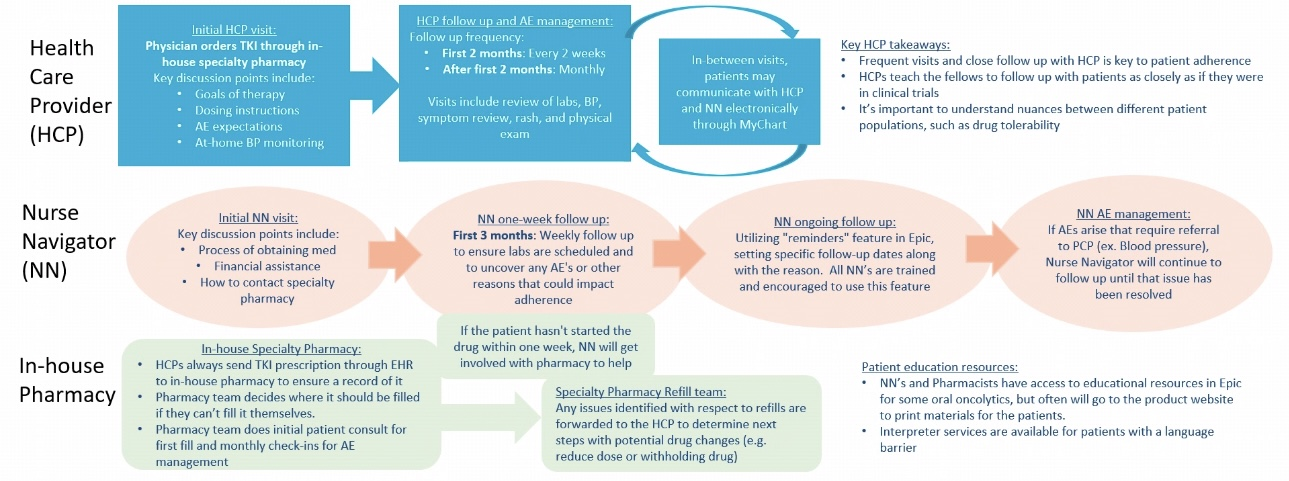

Supplement: sj-docx-1-onc-10.1177_11795549251341877 – Supplemental material for Evaluating Patient Adherence and Persistence to Tyrosine-Kinase Inhibitors for Metastatic Renal Cell Carcinoma: A Retrospective Analysis of Real-World Data [file sj-docx-1-onc-10.1177_11795549251341877.docx]
